# First-principles design of GaN–VHC (H = Cl, Br; C = Se, Te) van der Waals heterostructures for advanced optoelectronic applications†

**DOI:** 10.1039/d4ra08190k

**Published:** 2025-04-23

**Authors:** Sheraz Ahmad, Shah Saleem Ullah, Haleem Ud Din, Irina Piyanzina, Cuong Q. Nguyen

**Affiliations:** a School of Materials Science and Engineering, Nankai University Tianjin China; b Department of Physics, Hazara University Mansehra Pakistan; c Department of Physics, Bacha Khan University Charsadda Pakistan haleem.uddin@yahoo.com; d Center of Semiconductor Devices and Nanotechnology, Computational Materials Science Laboratory, Yerevan State University Republic of Armenia i.piyanzina@gmail.com; e Institute of Research and Development, Duy Tan University Da Nang 550000 Vietnam nguyenquangcuong3@duytan.edu.vn; f Faculty of Natural Sciences, Duy Tan University Da Nang 550000 Vietnam

## Abstract

In this study, we examine the structural, optoelectronic, and photocatalytic properties of GaN-based van der Waals heterostructures (vdWHs) that incorporate halogens (Cl, Br) and chalcogens (Se, Te). Using first-principles calculations based on density functional theory, we analyze six different stacking configurations of these heterostructures. Our results show that the GaN–VHC vdWHs (where H = Cl, Br and C = Se, Te) are both dynamically and energetically stable. For solar cell applications, the GaN–VHSe heterostructures exhibit a direct type-I band alignment, while the GaN–VHTe structures show an indirect type-I band alignment. All GaN–VHC heterostructures display strong optical peaks across the visible, infrared, and ultraviolet regions, highlighting their potential for optoelectronic applications. We investigated the photocatalytic potential of these heterostructures and found that GaN–VClSe performs water splitting at pH = 0. While model I and model II can facilitate water splitting, and mainly support reduction at pH = 0 and oxidation at pH = 7. However, their type-I band alignment inherently limits overall photocatalytic activity.

## Abstract 2

In this study, we investigated the structural, optoelectronic, and photocatalytic properties of GaN-based van der Waals heterostructures incorporating halogens (Cl, Br) and chalcogens (Se, Te).

## Graphical abstract

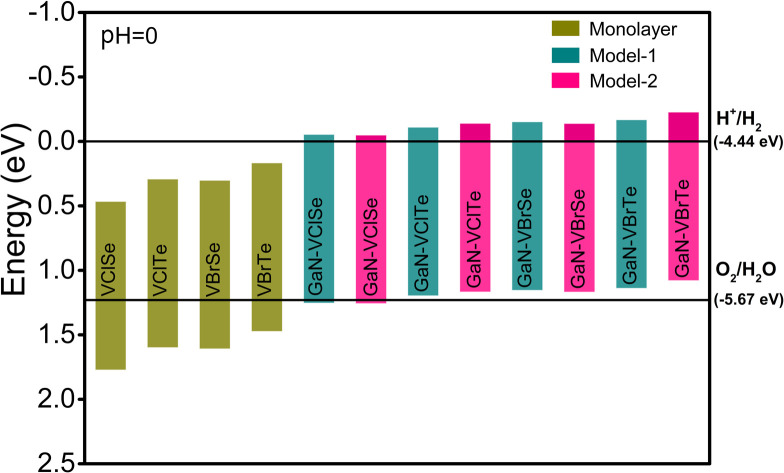

## 1 Introduction

There is a current focus on problems with the environment and the energy crises. Exploring renewable energy sources has gained a lot of interest in this context.^1^ Hydrogen (H_2_), which is necessary for enhanced energy conversion, is one of the primary sustainable sources of renewable energy.^2^ It has recently been shown that the sustainable extraction of H_2_ can be achieved by photocatalytic water splitting.^3^ It opens up potential for more affordable and effective H_2_ production techniques in addition to the use of conventional photocatalysts.^4,5^ Increasing a material’s efficiency and reaction rate is vital to the photocatalytic processes. Consequently, a keen interest in research has led to a search for materials with a high level of activity. Two-dimensional (2D) materials are currently a focus of considerable interest due to their many intrinsic characteristics, including their large surface area and inherent defects, enhancing the charge transfer and allowing for the modification of the electronic behavior.^6,7^ 2D materials are generally preferable to their 3D bulk nature forms because of these characteristics.^8,9^ Most significantly, 2D materials serve as the most available materials for determining how structure and photocatalytic performance are related.^10,11^

After the successful exfoliation of the 2D material graphene,^12^ the search for other two dimensional materials^13,14^ has been a focus of the research community to explore their astonishing properties. These 2D materials have a wide range of potential applications in light emitting devices,^15^ H_2_ storage,^16^ optoelectronics^17^ and photocatalytic water splitting to extract hydrogen.^18^ Recently, Janus transition metal dichalcogenides (JTMD) have drawn a lot of attention because of their unique features that make them different from their parent monolayers.^19–21^ JTMD have been synthesized using the chemical vapor deposition (CVD) method.^13^ Single layer VHC (H = Cl, Br; C = Se, Te) in the 2H phase, demonstrate stability and enhanced physical properties, indicating potential applications in valleytronics and spintronics.^22^ Two-dimensional binary compounds combine the characteristics of different monolayers. Equal numbers of individual atoms make up these materials. Recent studies have shown interest in group III–V binary compounds such as BN, AlN, and GaN because they have a similar number of valence electrons to graphene, show semiconducting properties, and are stable even in planar configuration.^23^ Theoretically, 2D h-AlN, h-GaN, and h-InN are considered promising candidates for electronic and optoelectronic devices due to their calculated wide bandgap, which can be either inherently direct or convertible to a direct bandgap, along with their high dielectric constant. Over the past two decades, gallium nitride (GaN) and related group III-nitrides have become critical materials for light-emitting diodes (LEDs) and laser diodes, driving significant advancements in GaN-based device technologies.^24^ Despite these developments, research efforts continue to enhance their performance further. GaN is particularly well suited for optoelectronic applications due to two key factors. First, its bandgap can be widely tuned across the ultraviolet (UV) and visible ranges by alloying with AlN and InN, respectively. Second, both n-type and p-type doping can be achieved by introducing Si and Mg impurities, enabling the formation of p–n junctions that facilitate efficient radiative recombination of electrons and holes. Beyond optoelectronics, GaN-based devices are also gaining traction in emerging fields such as solar cells, high-power transistors, and water-splitting technologies.^25^ A wide range of techniques, including atomic doping, strain application, alloying, and heterostructure formation, are employed to tailor the properties of two-dimensional layered materials.^26,27^

A variety of methods, such as doping, alloying, applying strain, forming super lattices, and layer stacking, are frequently used to alter the physical characteristics of 2D materials. One of the best ways to adjust material properties is to stack monolayers to create van der Waals heterostructures (vdWHs), which give precise control over the size, shape, and synergistic combination of the individual monolayer characteristics. vdWHs are generally classified into type-I, type-II, and type-III band alignments, depending on the relative positions of the valence band (VB) and conduction band (CB) edges of the constituent monolayers near the Fermi level.^28^ Among these various methods, the 2D monolayers are vertically stacked on top of each other and are held together by van der Waals interactions to form heterostructures (vdWHs). This is an effective technique to alter the properties of individual monolayers and the resultant heterostructure inherits the combined properties of the individual monolayers. Depending on the orientation of the conduction and valence band edges of each monolayer near the Fermi level, these vdWHs are usually considered to possess type-I, type-II and type-III band alignments.^29^ In type-I (type-II) band gap semiconductors the Fermi level is contributed by both the valence (VB) and conduction bands (CB) in the same (different) monolayers making the material suitable for photovoltaic applications. In type-III broken band alignment either the CB or VB exceeds the Fermi level.^30^ 2D materials having a staggered band structure (type II) are suitable to effectively separate the charge carriers in the different constituents, thereby making them promising for application in photocatalytic water spitting.^31^ Experimental and theoretical studies on these types of 2D materials have shown their application to photocatalytic water splitting.^32^ Using first-principles calculations, Idrees *et al.* examined the structural, optoelectronic, and photocatalytic characteristics of GeC–VXY (X = Cl, Br; Y = Se, Te) vdWHs. Their findings support dynamic and energetic stability, and in certain situations, type-I band alignment indicates laser applications, while type-II band alignment makes them appropriate for solar cell applications. Effective electron–hole separation is supported by charge transfer analysis, and exciton-dominated transitions are revealed by optical properties. Lastly, model-II vdWHs have the potential to produce clean energy since photocatalytic studies show they can split water.^22^

In this work, a study of vdWHs made of GaN and VHC (H = Cl, Br; C = Se, Te) monolayers is carried out by using density functional theory based first principles calculations. This research is motivated by the adequate lattice mismatch and high energetic feasibility. Based on the alternate attachment of the H (halogens) and C (chalcogens) atoms in the top and bottom layers, two models of GaN–VHC vdWHs are created. The stability of the structures is confirmed by calculating the thermal and dynamic stability. Electronic properties including interlayer charge transfer and work functions are studied. Furthermore, the suitability of GaN–VHC vdWHs is confirmed by exploring optical and photocatalytic properties.

## 2 Computational details

In the current research work density functional theory in combination with the projected augmented wave (PAW) method,^33^ implemented in VASP, is employed to perform first principles calculations.^34^ The DFT-D3 Grimme approach^35^ is employed for weak vdW correction within the Perdew–Burke–Ernzerhof (PBE) function based on the generalized gradient approximation (GGA).^36^ A cutoff energy of 500 eV for the plane wave basis set and Monkhorst–Pack grid of 12 × 12 × 1 *k*-points mesh in the first Brillouin zone (BZ) are used. Total energy convergence and forces are set to 10^−5^ eV and 0.01 eV Å^−1^. In order to avoid any periodic interaction, a 25 Å vacuum is introduced along the *z* direction. In order to gain a better understanding of the band structure, we employ the screened hybrid Heyd–Scuseria–Ernzerhof (HSE06) method,^37,38^ since the PBE function and GGA approach typically underestimate the band gap. Moreover, the GW0 approach is used to solve the Bethe–Salpeter equation (BSE) to calculate the optical properties.^39,40^ In order to verify the thermal stability of the studied vdWH, we employed *ab initio* molecular dynamics (AIMD) simulation^41,42^ at 500 K temperature for a total time of 5 ps with a 1 fs time interval using the Nosé–Hoover thermostat method.

## 3 Results and discussion

**Fig. 1:**
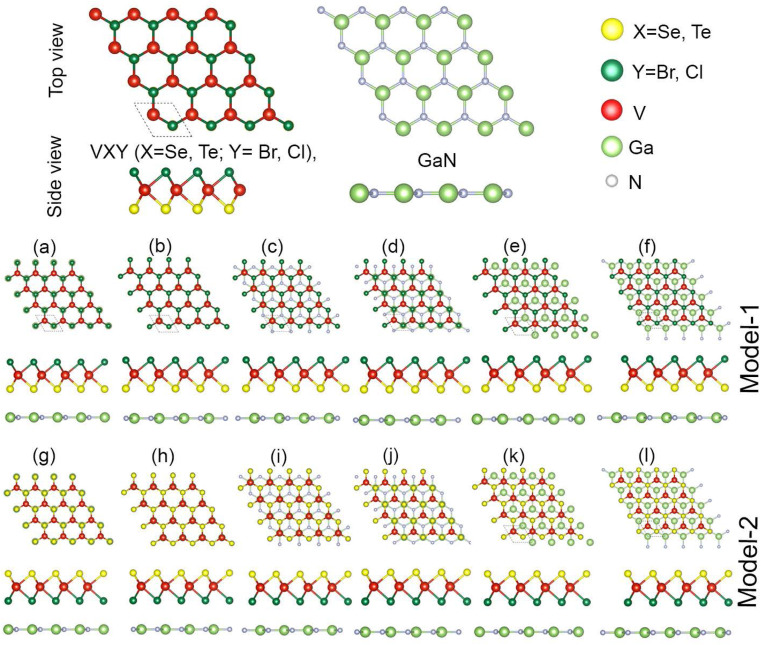
The atomic structures of the VHC (H = Br, Cl; C = Se, Te) monolayer and GaN monolayer. Row 1 (a–f) contains the different stacking configurations of the model-I GaN–VHC heterostructures; row 2 (g–l) contains model-II stacking configurations.

Here, we have two terminated surfaces in VHC monolayers with two different chalcogenide Se and Te atoms bonded on either side of the V atom. These surfaces are reactive to other layers, thereby making them suitable to create vdWHs of GaN and VHC monolayers. The combinations of the GaN and VHC monolayers include two different models, namely model-I and model-II, as depicted in Fig. 1. In model I, the chalcogen atom is positioned on the lower side of the monolayer, facing the GaN monolayer, while in model II, the halogen atoms occupy this position. Each model of the GaN–VHC (H = Cl, Br; C = Se, Te) vdWHs is formed with six possible atomic stacking configurations (i–vi), as shown in Fig. 1. In the first stacking arrangement (a), the chalcogen atom is aligned with the Ga atom, whereas in (b), it is positioned above the N atom. In configuration (c), the chalcogen atom is located at the center of the GaN hexagon. Similarly, in (d), the V atom is placed at the center of the GaN hexagon, while in (e), it is aligned with the N atom. Finally, in stacking (f), the V atom is positioned at the center of the hexagonal GaN monolayer. The geometric properties of the GaN and VHC (H = Cl, Br; C = Se, Te) monolayers were first determined and are shown in Table S1 of the ESI.† These values agree well with previously known data^43^ demonstrating the accuracy of our calculations. The small, experimentally achievable lattice mismatch, coupled with the identical hexagonal symmetry, observed for designing GaN–VHC (H = Cl, Br; C = Se, Te) vdWHs, indicates their suitability for experimental synthesis.^44^

In general, the local layer stacking configuration and relative layers orientation on top of each other have a considerable impact on the interfacial characteristics of the vdWH. The most energetically favorable configuration of model-I (model-II) is determined by calculating the interlayer separation *d* and binding energy using *E*_b_ = *E*_GaN–VHC_ − *E*_GaN_ − *E*_VHC_, where *E*_GaN–VHC_ and *E*_GaN_ (*E*_VHC_) are the total energies of the GaN–VHC vdWH and individual GaN (VHC) monolayers, respectively. These values are listed in Table 1. High energetic stability and strong physical interaction between the GaN and VHC monolayers in these stacking configurations will be demonstrated by minimum binding energy and reduced interlayer separation. Stacking configuration (iii) for model-I and (i) for model II have the lowest binding energies and shortest interlayer separation, so they are energetically the most stable configurations. Estimated binding energies and smaller interlayer spacing agree well with previously calculated results.^32^

**Table 1:** Calculated band gap (*E*_g_) given by PBE and HSE functionals, interlayer distance (*d*), lattice constant (*a*), work function (*Φ*) and charge transfer (Δ*Q*) in the GaN–VHC heterostructures

Model	Heterostructure	*E*_g_ (eV)/nature		*d* (Å)	*a* (Å)	*Φ* (Å)	Δ*Q* (e)
		PBE	HSE				
Model-I	GaN–VClSe	0.86/indirect	1.33/direct	3.32	3.21	5.07	0.016
	GaN–VClTe	0.93/indirect	1.30/indirect	3.20	3.26	4.55	0.015
	GaN–VBrSe	0.76/indirect	1.34/direct	3.41	3.25	5.08	0.011
	GaN–VBrTe	0.69/indirect	1.24/indirect	3.36	3.30	4.36	0.011

Model-II	GaN–VClSe	0.70/indirect	1.31/direct	3.14	3.21	5.35	0.014
	GaN–VClTe	0.94/indirect	1.36/indirect	3.33	3.26	4.74	0.003
	GaN–VBrSe	0.53/indirect	1.32/indirect	3.09	3.25	5.34	0.023
	GaN–VBrTe	0.79/indirect	1.36/direct	3.29	3.30	4.75	0.001

**Fig. 2:**
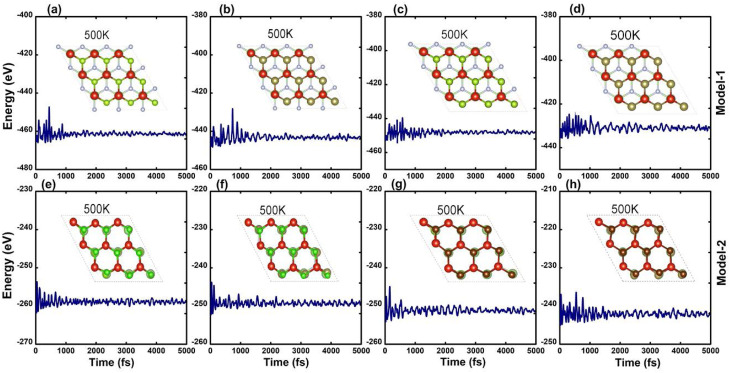
AIMD simulations for model-I (a–d) and model-II (e–h) (GaN–VClSe, GaN–VClTe, GaN–VBrSe, GaN–VBrTe) at 500 K.

**Fig. 3:**
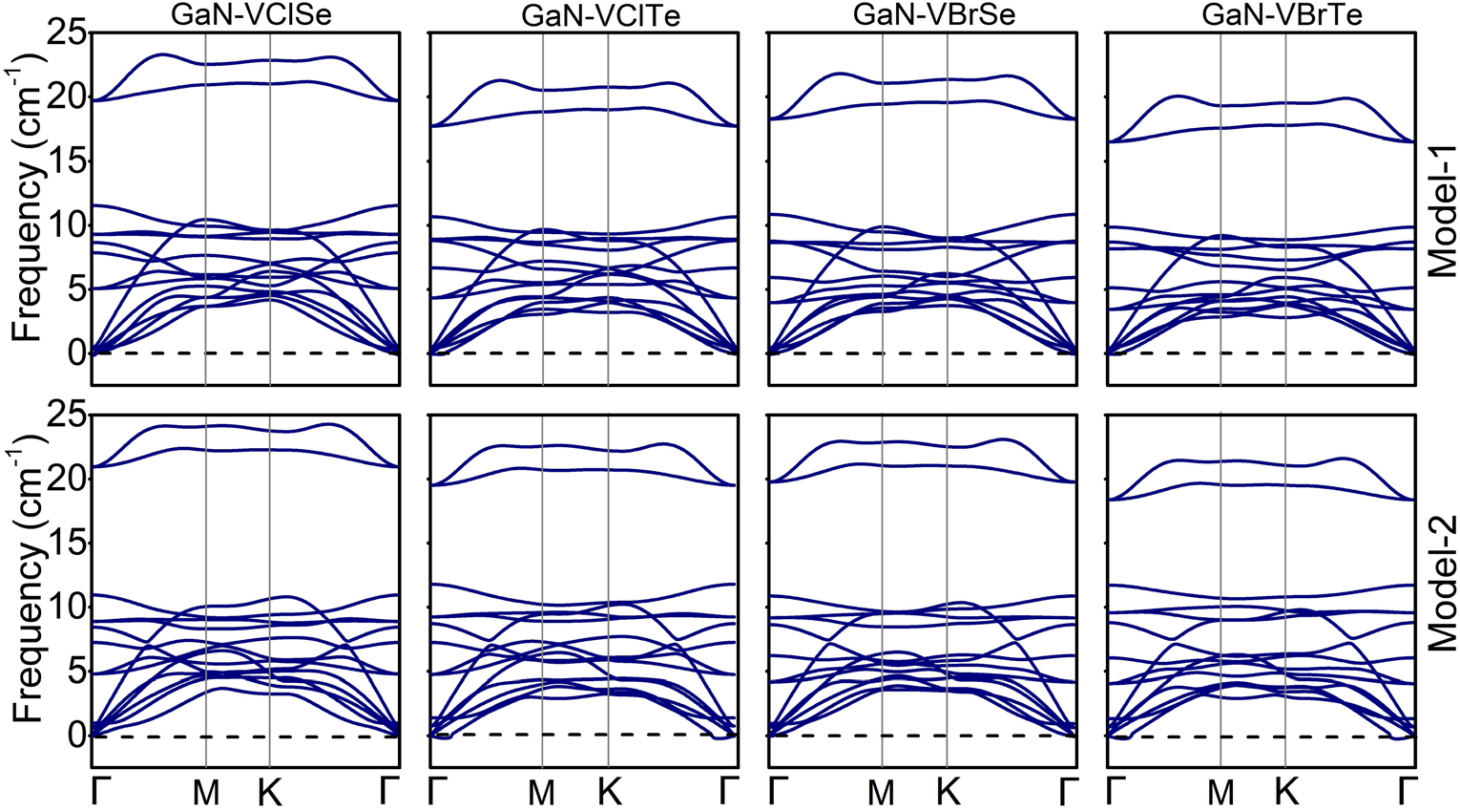
Phonon spectra for model-I (row-1) and model-II (row-2) of GaN–VClSe, GaN–VClTe, GaN–VBrSe, GaN–VBrTe.

Furthermore, thermal stability of the energetically most stable stacking of GaN–VHC (H = Cl, Br; C = Se, Te) vdWHs is predicted by using AIMD simulation, which is an important indicator to design 2D materials and their vdWHs. Thus, the thermal stability of the GaN–VHC vdWHs in the stacking (iii) for model-I and stacking (i) for model-II is considered, as depicted in Fig. 2. It is simple to verify that the GaN–VHC vdWHs maintain their atomic structures with no distortions. In addition, the fluctuation in the total energies is small. All these findings confirm that both models of studied vdWHs are thermally stable at the temperature (500 K). Further calculations are performed for stacking (iii) in model-I and stacking (i) in model-II. Additionally, the phonon spectra in Fig. 3 of all the GaN–VHC vdWHs are also illustrated to investigate their stability. A minor imaginary frequency was observed near the *Γ* point. This occurrence does not indicate structural instability but may instead result from numerical instability in accurately calculating rapidly diminishing interatomic forces. The observed imaginary frequency modes in heterostructures such as GaN–VClTe and GaN–VBrTe likely arise from very small negative frequencies or flexible modes associated with van der Waals interactions between layers. These modes do not signify substantial instability but rather reflect the subtle balance of forces within these layered structures. One can observe that all the GaN–VHC vdWHs do not exhibit any imaginary frequencies at the *Γ* point, as shown in Fig. 3. This finding demonstrates that all the GaN–VHC vdWHs are dynamically stable at room temperature.

**Fig. 4:**
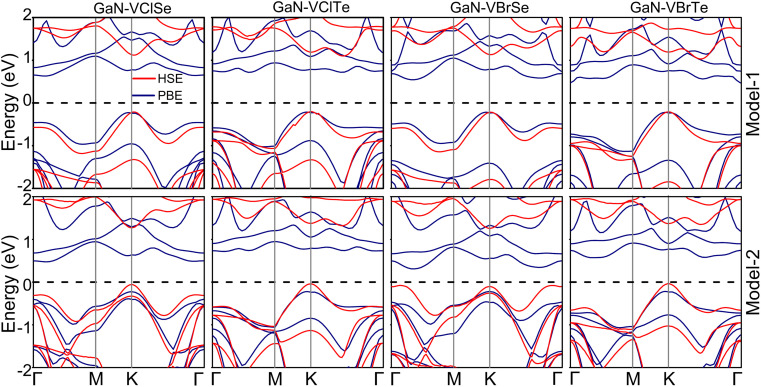
PBE and HSE band structures for model-I (row-1) and model-II (row-2) of the GaN–VHC heterostructures, where H = Cl, Br and C = Se, Te.

**Fig. 5:**
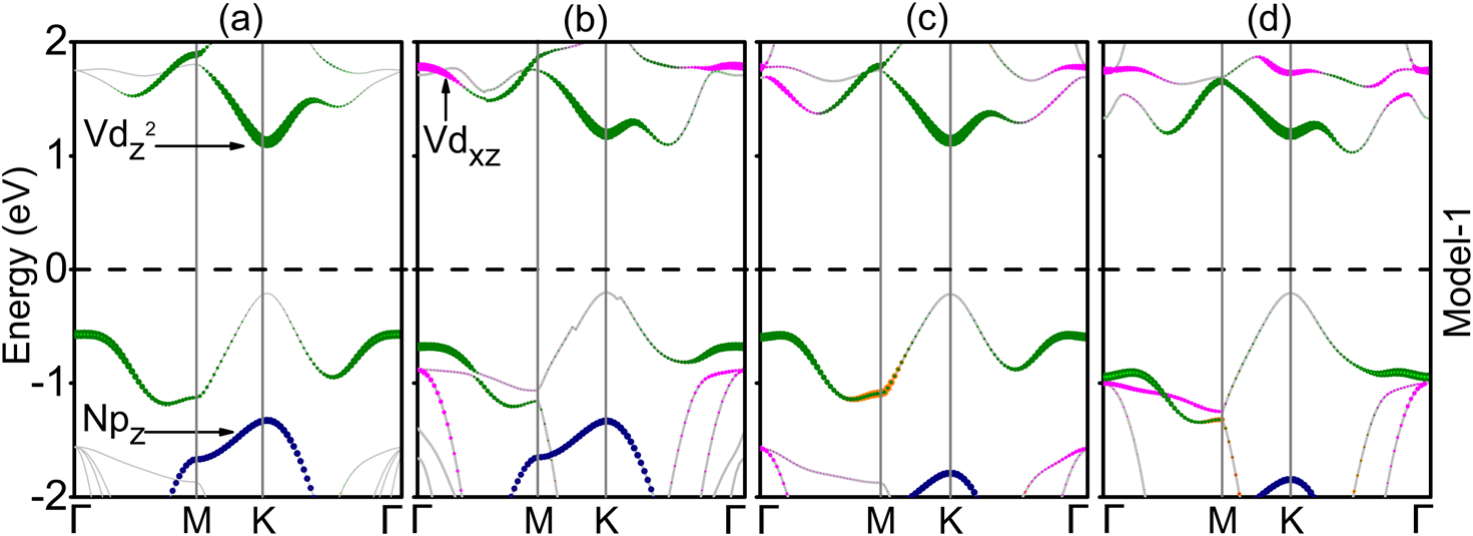
Weighted band structures of GaN–VHC (H = Cl, Br; C = Se, Te) of model-I; (a and c) direct and (b and d) indirect band gap semiconductors.

Furthermore, the choice of exchange correlation function has a significant impact on the electronic characteristics of materials. Hence, we took into account both the PBE and HSE06 functionals to calculate the band structures of GaN–VHC vdWHs for both model-I and model-II. These results are listed in Table 1 and Fig. 4. We find that the GaN–VClSe (VBrSe) vdWH for both the model I and II exhibits semiconducting behavior with a direct band gap. The band edges of such vdWHs are located at the *K* point, as depicted in Fig. 4. In contrast, model-I of the GaN–VClTe (VBrTe) vdWH is an indirect semiconductor with the VBM at the *K* point and the CBM along the *K*–*Γ* path, while the model II is a direct semiconductor. The band gap values estimated using the PBE and HSE06 approaches are presented in Table 1. Since the PBE technique underestimates the band gap, it is evident that HSE06 band gaps are greater than those predicted by the PBE method. It is noted that the same vdWH in different models has different band gap values due to the attachment of chalcogenide atoms to different sides of the VHC monolayer. In all the studied vdWHs of both model-I and model-II there will be no rapid recombination of photogenerated charge carriers and they are suitable for charge carrier separation which leads to their application in photocatalytic water splitting.^45^ Additionally, we find that the generation of the GaN–VHC vdWHs leads to tunable band gaps compared to the constituent monolayer, as listed in Tables S1 and S2 of the ESI.† Furthermore, the projections of each layer to the vdWHs are examined by considering the weighted band structures of GaN–VHC vdWHs, as illustrated in Fig. 5.

Interestingly, in the GaN–VClSe for both models and in the GaN–VBrSe for model I, the V-d_*z*^2^_ atom of the VHC layer is responsible for the contribution to the VBM at the *K*-point, while the V-d_*z*^2^_ state of the VClSe (VBrSe) layer contributed mainly to the CBM at the *K*-point. This confirms the type-I band alignment for these vdWHs. Without the use of an external electric field, these localizations occurred in the type-I band alignment in the VBM (CBM) from the identical monolayers. This intrinsic electric field may be created by bond bending, which causes GaN–VClSe (GaN–VBrSe) to become a vdWH. This inherent field causes photogenerated electrons to go in distinct directions. As a result, the increased electron–hole pairs substantially shorten the recombination period, which has interesting applications in light harvesting and detection. In addition, the remaining vdW GaN–VClTe (GaN–VBrTe) heterostructure exhibits type-I band alignment, where the VBM is located at the *K* point from the V-d_*z*^2^_ state of the VHC layer and the CBM lies between the *K* and *Γ* points giving them an indirect type-I band alignment. This alignment is advantageous for the design of light-emitting diodes and lasers. In addition, type-I band alignment may not provide the optimal advantages for solar cell applications. However, type-I alignment typically presents challenges for efficient charge separation. Our findings suggest that the specific characteristics of the GaN-based vdW heterostructures may still offer unique pathways for energy conversion.

**Fig. 6:**
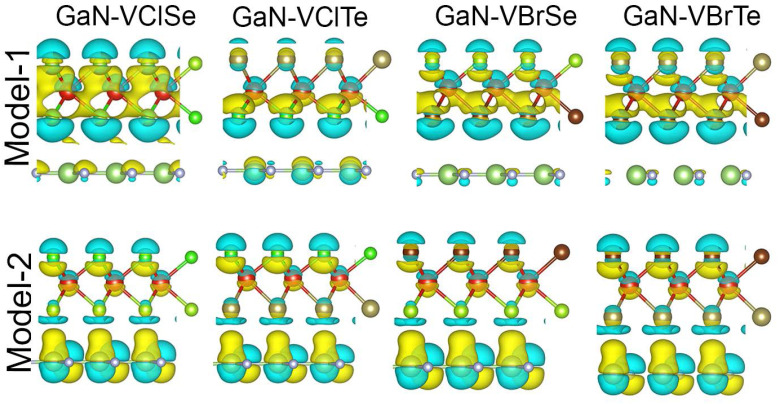
Charge density difference for GaN–VHC heterostructures (H = Cl, Br; C = Se, Te) where row-1 is for model-I and row-2 is for model-II.

Furthermore, we examine the charge transfer between two layers by analyzing the charge density difference (CDD) as below:

1 Δ*ρ* = *ρ*_GaN–VHC_ − *ρ*_GaN_ − *ρ*_VHC_

where *ρ*_GaN–VHC_ represents the overall charge density of GaN–VHC (H = Cl, Br; C = Se, Te) vdWHs and *ρ*_GaN_ (*ρ*_VHC_) represents the individual GaN (VHC) charge densities, as shown in Fig. 6. The inter-layer charge transition in vdWHs is shown by CDD and Bader charge analysis. As can be seen from Fig. 6, most of the charge is transferred from the VHSe layer to the GaN monolayer at the GaN–VClSe (GaN–VBrSe) vdWH interface for both models, but there is a modest contact between VHTe and GaN in GaN–VClTe (GaN–VBrTe), as has already been shown in GeC–MSSe.^46^ We additionally examined the potential drop (Δ*V*) across the GaN–VHC vdWHs. These results showed that single layers of GaN and VHC monolayers may exhibit distinct excitonic behaviors from heterostructures, aiding in the electron and hole separation process, as shown in Fig. 6 and Table 1. By varying the work function (*Φ*), the vdWHs of the two monolayers can be improved, enhancing the electrical characteristics of these systems. Charges are transferred from the GaN layer to the VHTe monolayer. In Fig. 6, the yellow color indicates electron increase while the cyan color represents electron depletion. Therefore, it can be deduced that upon forming the GaN–VHSe (Te) vdWHs, VHSe (GaN) undergoes n-doping, whereas GaN (VHTe) undergoes p-doping at the GaN–VHC vdWHs interface.

**Fig. 7:**
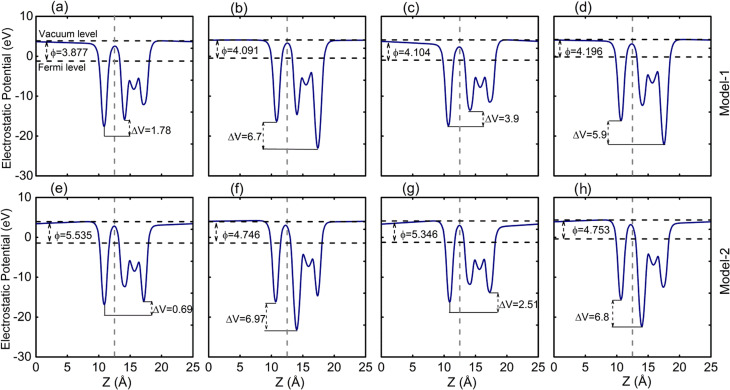
Planar-average electrostatic potential of GaN–VHC (H = Cl, Br; C = Se, Te) of (a–d) model-I and (e–h) model-II.

To quantify the charge transfer between the VHC and the GaN layer, we performed Bader charge analysis. For model-I, the analysis showed that 0.016, 0.015 (0.011, 0.011) *e* per unit cell are transferred from the VHC (H = Cl, Br; C = Se, Te) to the GaN layer. Similarly, for model-II, we found charge transfers of 0.014, 0.003 (0.023, 0.0016) *e* per unit cell are transferred from the VHC (H = Cl, Br; C = Se, Te) to the GaN layer. These minor charge transfers from one layer to the other confirm the weak vdW interactions between these layers. To further verify the charge transfer, we examine the electrostatic potential of GaN–VHC vdWHs along the *z*-axis, which is shown in Fig. 7. It is evident that the GaN (VHTe) monolayer has a deeper potential than that of the VHSe (GaN) layer, confirming the charge transfer.

**Fig. 8:**
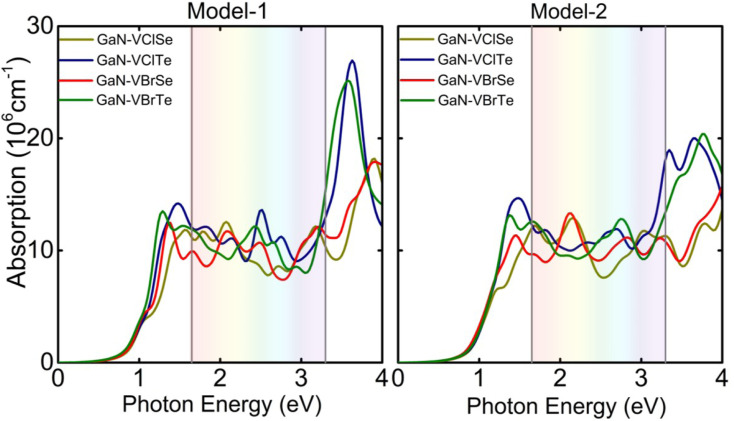
Optical absorption spectra for GaN–VHC (H = Cl, Br; C = Se, Te) for model-I and model-II.

The capacity to absorb visible light is a crucial need for an effective photocatalyst. The optical absorption coefficient is used to estimate the amount of light with a specific energy or wavelength that can pass through a material before being absorbed. So, a substance with a greater absorption coefficient will absorb photons more quickly. Mathematically, a material’s absorption coefficient *α*(*ω*) can be written as:2
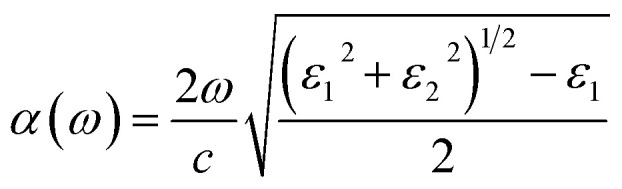
where *ε*_1_ and *ε*_2_ are the real and imaginary halves of the dielectric function, respectively, and denote the frequency of light. The optical absorption coefficient is boosted by the decay of the light intensity dispersion across the medium unit length. The absorption coefficients of heterostructures are determined, as shown in Fig. 8, to verify the reliability of our methodology. The solar light’s first maximum absorption peak, which occurs in the infrared region, is remarkably consistent with previous research.^47^ Additionally, a built-in electric field caused by the charge transfer between the GaN layer and the VHC divides the photo-generated charge carriers in various constituents spatially.

The initial excitonic peak is the one we are interested in. The initial peaks for model-I of GaN–VClSe (VClTe) and GaN–VBrSe (VBrTe) and are located at 1.56 (1.48) eV and 1.38 (1.31) eV, respectively. On the other hand, the first peaks for GaN–VClSe (VClTe), GaN–VBrSe (VBrTe) vdWHs are located at 1.25 (1.48) and 1.45 (1.37) eV for model II, respectively. By constructing the vdWHs, we can observe that there are blue shifts in the excitonic peaks which shift to higher energy.^48^ All of the investigated systems exhibit high peaks in the visible range, which makes GaN–VHC vdWHs a promising choice for electronic and optoelectronic device applications. High optical absorption requires a large peak that occupies a high number of states at the Fermi level.^49^ In addition, we computed the excitonic binding energies of GaN–VHC vdWHs, which are −0.76 (−0.81) eV for GaN–VClSe, and −0.86 (−0.911) for GaN–VClTe, −0.79 (−0.84) for GaN–VBrSe and −0.85 (−0.90) for GaN–VBrTe in model-I (model-II), respectively. The GaN–VHSe (GaN–VHTe) in model-I (model-II) also showed a red (blue) shift, which could be brought on by the various chalcogen atoms affixed to the GaN layer.

**Fig. 9:**
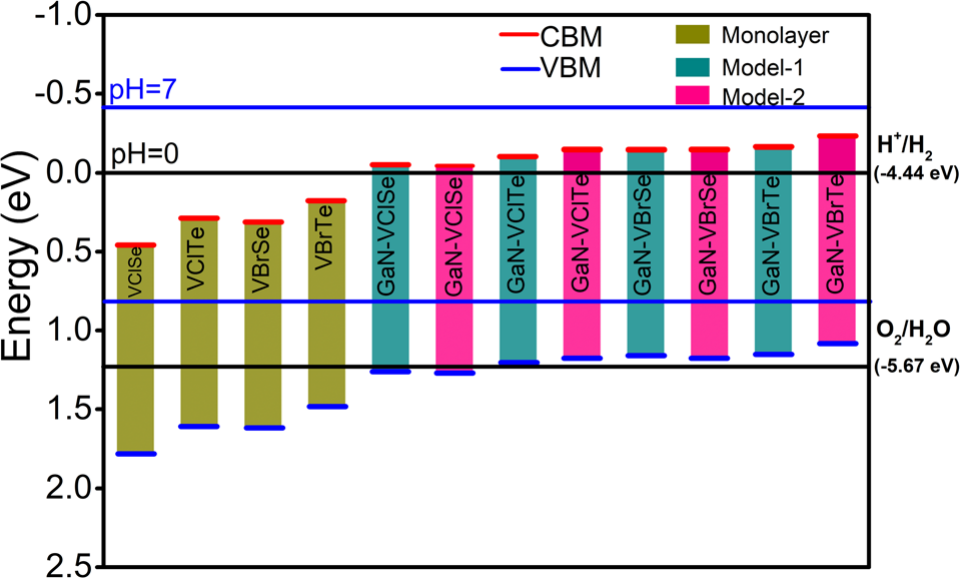
Valence and conduction band edges potentials of GaN–VHC vdWHs, where green bars represent monolayers and cyan and pink bars represent conduction and valence band edge positions for the standard reduction (oxidation) potentials, −4.44 eV (−5.67 eV), for water splitting.

Using the Mulliken electronegativity, the band edge positions of the VHC monolayers and GaN–VHC vdWHs are examined with regard to the standard reduction (oxidation) potential of −4.44 eV (−5.67 eV) for full water splitting at pH = 0, as presented in Fig. 9. It is evident that only VHC monolayers cannot reduce water to O_2_/H_2_O, but can successfully oxidize it to O_2_. GaN–VClSe vdWH model-I (model-II) could be a promising candidate for photocatalytic water splitting at pH = 0, as it has an energy level for the CBM (VBM) that is notably higher than that of the standard redox potentials. This energy is sufficient to drive photogenerated holes and electrons to split water into O_2_/H_2_O and H^+^/H_2_. When it comes to model-I (model-II) GaN–VHC vdWHs, the VBM edge is higher than the oxidation potential except for GaN–VClSe for both model-I and model-II, which prevents water from being oxidized, whereas the CBM straddles the reduction potential and responds well to splitting water into H^+^/H_2_. To gain a deeper comprehension of the photocatalytic performance for water splitting, it is imperative to identify the significant band edge positions,^49^ as depicted in Fig. 9. The GaN–VHC heterostructures band edge positions for model I (and model II) are computed using the HSE06 function. Typically, pH levels have an impact on the oxidation (*E*_ox_ = −5.67 eV + pH × 0.059 eV) and reduction (*E*_red_ = −4.44 eV + pH × 0.059 eV).^50,51^ For water splitting at pH = 0 (pH = 7), the conventional reduction and oxidation potentials are therefore −4.44 (−4.03) eV and −5.67 (−5.26) eV, respectively. It is evident that at pH = 0, all pure monolayers have reasonable energy levels for the valence band (VB) and conduction band (CB) edge positions for redox potentials. Additionally, when pH = 7, the VB and CB edge positions are advantageous for the dissociation of water into H^+^/H_2_ and O_2_/H_2_O. However, due to the short lifetime (3–10 ps) of the photogenerated charge carriers, single semiconductors are rarely used as photocatalysts for the water dissociation event. Rather, by triggering redox processes in various layers, the design of a vertically stacked heterojunction can successfully extend the lifetime of a charge carrier.^52^

The CB edge of the GaN–VHC system can reduce water into H^+^/H_2_ and is positioned above (below) the typical reduction potential at pH = 0 (pH = 7). Nevertheless, at pH = 0 (pH = 7), the VB edge is located above (below) the normal oxidation potential, allowing (not allowing) water to oxidize into O_2_/H_2_O. More intriguingly, GaN–VClSe heterostructures may appear to be capable of water dissociation into H^+^/H_2_ and O_2_/H_2_O at pH = 0 due to their appropriate band edge positions for redox potentials. Fig. 9 illustrates that, aside from GaN–VClSe, all other heterostructures are capable of oxidation but not of water reduction at pH = 7 and while capable of reduction at pH = 0, are unable to convert water into H^+^/H_2_ at pH = 7. It is evident that GaN–VHC model-I and model-II both could have a better reaction to photocatalytic response than other heterostructures. Therefore, by creating vdWHs with the GaN layer, it confirms that the photocatalytic process is particularly sensitive to the relative order of chalcogen atoms in the VHC layer. Comparable patterns are also shown for TMDC–TMDC vdWHs and GeC–MSSe.^43^ Our findings indicate that GaN–VClSe vdWHs model-I and model-II have potential for low-cost, large-scale solar hydrogen production. However, their type-I band alignment limits full water splitting, restricting their overall photocatalytic efficiency.

## 4 Conclusions

In conclusion, the structural, electronic, optical and photocatalytic features of GaN–VHC (H = Cl, Br; C = Se, Te) vdWHs are examined by the use of first principles computation. Both models of GaN–VHC (H = Cl, Br; C = Se, Te) vdWHs are energetically and dynamically stable, according to our computations. Direct (indirect) type-I band alignment is demonstrated by GaN–VHSe (GaN–VHTe) vdWHs for models I and II, which is extremely desirable for solar cell applications. The charge transfer in these vdWHs is further confirmed by electrostatic potential, charge density difference, and Bader charge analysis. The imaginary part of the dielectric function is calculated for both model-I and model-II to understand the optical behavior of these systems, where the lowest energy excitations in both models are dominated by excitonic transitions. All the studied heterostructures GaN–VHC (H = Cl, Br; C = Se, Te) exhibit strong peaks in the visible, infrared, and ultraviolet regions, indicating their potential for use in optoelectronic applications. Although model-I and model-II can facilitate water splitting into O_2_/H_2_O and H^+^/H_2_, the overall photocatalytic response showed that only GaN–VClSe achieved full water splitting at pH = 0. However, all proposed heterostructures, including GaN–VClSe, exhibit type-I band alignment, which limits their ability to sustain overall water splitting.

## Data availability

The data that support the findings of this study are available from the corresponding author upon reasonable request.

## Conflicts of interest

There are no conflicts to declare.

## Supplementary Material

RA-015-D4RA08190K-s001
