# Supplementary material for: First-principles design of GaN–VHC (H = Cl, Br; C = Se, Te) van der Waals heterostructures for advanced optoelectronic applications
Source: RSC Adv. 2025 Apr 23;15(17):13076–85. doi: 10.1039/d4ra08190k (PMC12016790; doi:10.1039/d4ra08190k)
Supplement: RA-015-D4RA08190K-s001 [file RA-015-D4RA08190K-s001.pdf]

Supplementary Information (SI) for RSC Advances.
This journal is © The Royal Society of Chemistry 2025

Supporting Information (SI)
First-Principles Design of GaN-VHC (H = Cl, Br; C = Se, Te) van der Waals Heterostructures for Advanced Optoelectronic Applications

Sheraz Ahmad[1], Shah Saleem Ullah[2], Haleem Ud Din[3*], Irina Piyanzina[4*], Cuong Q. Nguyen[5,6,*]

[1]School of Materials Science and Engineering, Nankai University, Tianjin, China.
[2]Department of Physics, Hazara University, Mansehra Pakistan.
[3]Department of Physics, Bacha Khan University, Charsadda, Pakistan.
[4]Center of semiconductor devices and Nanotechnology, Computational Materials Science Laboratory, Yerevan State University, Republic of Armenia.
[5]Institute of Research and Development Duy Tan University, Da Nang 550000 Vietnam
[6]Faculty of Natural Sciences, Duy Tan University, Da Nang, 550000 Vietnam

Email: H. U. Din (haleem.uddin@yahoo.com), C. Q. Nguyen (nguyenquangcuong3@duytan.edu.vn) and Irina Piyanzina (i.piyanzina@gmail.com)

Table S1. The optimized lattice constant, bond length, and band gap PBE (E_g) and HSE06 (E_g) values of the GaN and VHC (H= Cl, Br; C=Se, Te) monolayers.

	**GaN**	**VClSe**	**VClTe**	**VBrSe**	**VBrTe**
Band (PBE)	1.85 Indirect	0.843 Indirect	0.90 Indirect	0.75 Indirect	0.75 Indirect
Band (HSE06)	3.19 Indirect	1.31 direct	1.40 direct	1.31 direct	1.33 Indirect
a (Å)	3.25	3.21	3.26	3.25	3.3
V-X (Å)	---	2.46	2.64	2.47	2.64
V-Y (Å)	---	2.41	2.41	2.52	2.52
Ga-N (Å)	1.87	---	---	---	---
Angle (θ) X-V-Y	120	81.07	83.37	82.67	85.10

Table S2. Shows binding energies E_b (eV) of different staking configurations of model-1 and model-2 for GaN-VHC (H=Cl, Br; C=Se, Te) heterostructures.

	Model-1				Model-2			
	GaN-VClSe	GaN-VClTe	GaN-VBrSe	GaN-VBrTe	GaN-VClSe	GaN-VClTe	GaN-VBrSe	GaN-VBrTe
1	-0.72 eV	-0.80 eV	-0.74 eV	-0.78 eV	-0.79 eV	-0.86 eV	-0.81 eV	-0.84 eV
2	-0.69 eV	-0.76 eV	-0.71 eV	-0.75 eV	-0.72 eV	-0.79 eV	-0.73 eV	2.58 eV
3	-0.73 eV	-0.81 eV	-0.75 eV	-0.79 eV	-0.77 eV	-0.84 eV	-0.79 eV	2.40 eV
4	5.32 eV	4.47 eV	5.61 eV	4.48 eV	-0.78 eV	-0.84 eV	1.06 eV	0.18 eV
5	4.29 eV	0.79 eV	4.26 eV	3.48 eV	-0.77 eV	-0.85 eV	0.58 eV	0.18 eV
6	5.32 eV	4.47 eV	5.61 eV	4.48 eV	-0.72 eV	-0.79 eV	1.06 eV	-0.76 eV

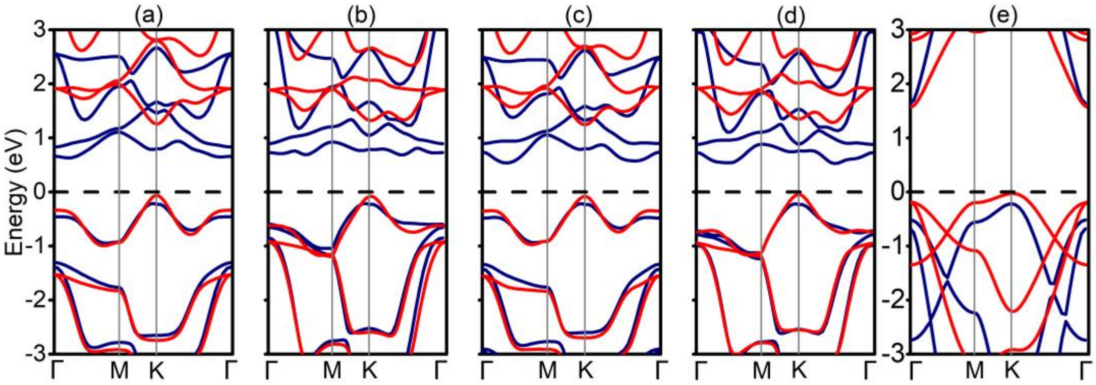

Figure S1. PBE (blue) and HSE (red) band structures for monolayers (a-e) ClSe, ClTe, BrSe, BrTe and GaN.

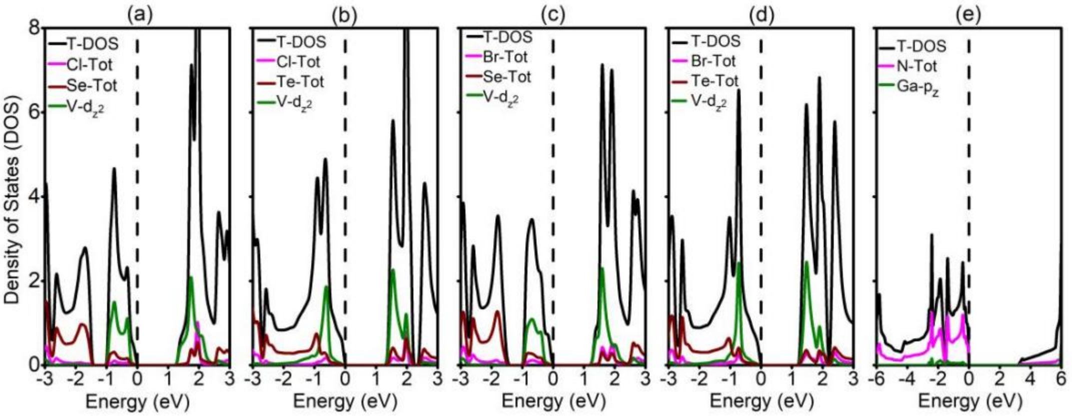

Figure S2. Partial Density of States for monolayers (a-e) ClSe, ClTe, BrSe, BrTe and GaN.

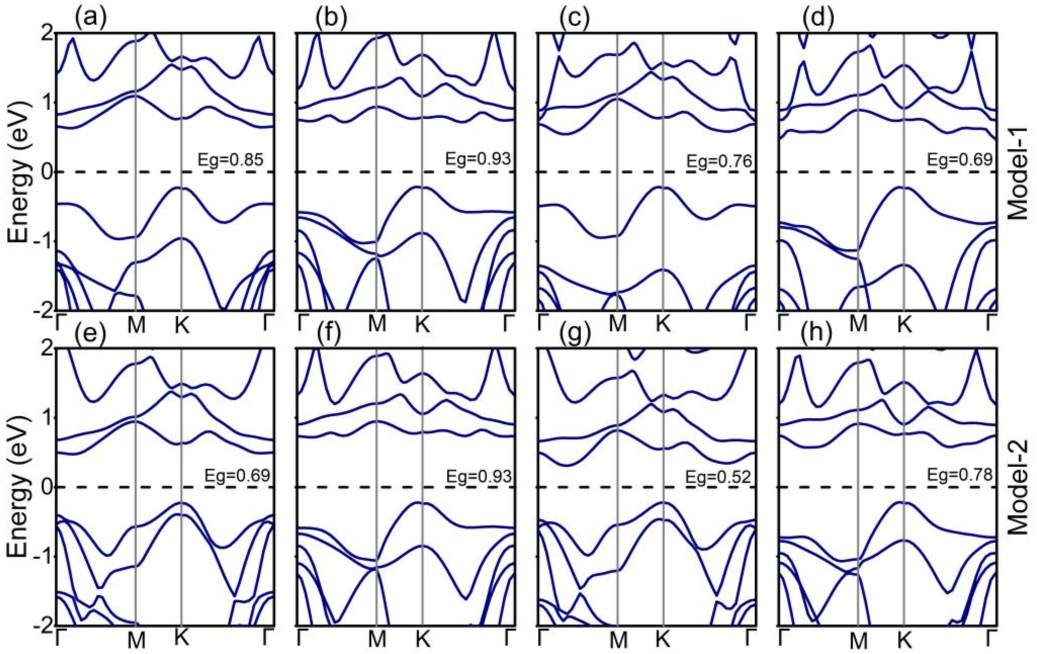

Figure S3. PBE Band structure for Model-1 and Model-2 for (GaN-VClSe, GaN-VClTe, GaN-VBrSe, GaN-VBrTe)

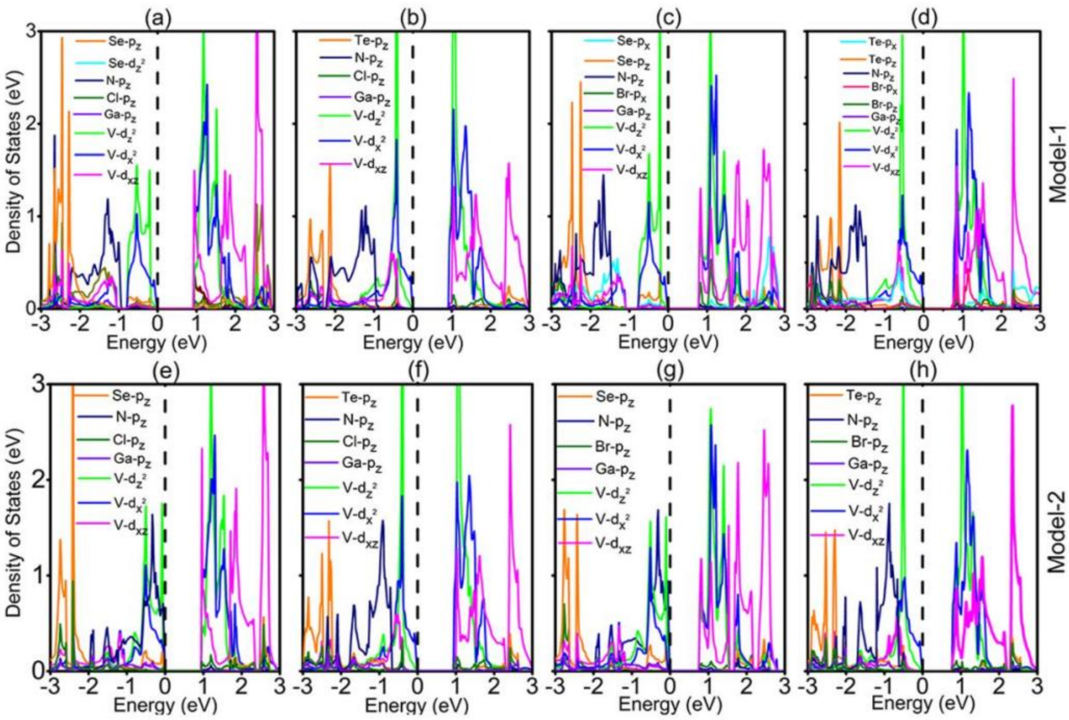

Figure S4. Partial density of states (PDOS) for first row model 1 and second row model-2 of (GaN-VClSe, GaN-VClTe, GaN-VBrSe, GaN-VBrTe)
